# Creation of a Stable Nanofibrillar Scaffold Composed of Star-Shaped PLA Network Using Sol-Gel Process during Electrospinning

**DOI:** 10.3390/molecules27134154

**Published:** 2022-06-28

**Authors:** Karima Belabbes, Coline Pinese, Christopher Yusef Leon-Valdivieso, Audrey Bethry, Xavier Garric

**Affiliations:** 1 Polymers for Health and Biomaterials, IBMM, CNRS, ENSCM, University of Montpellier, 34090 Montpellier, France; belabbeskarimapharm@gmail.com (K.B.); christopher-yusef.leon-valdivieso@umontpellier.fr (C.Y.L.-V.); audrey.bethry@umontpellier.fr (A.B.); xavier.garric@umontpellier.fr (X.G.); 2 Department of Pharmacy, Nîmes University Hospital, 30900 Nimes, France

**Keywords:** functionalized polymers, silylated PLA, crosslinking in situ, hybrid network, soft tissues regeneration

## Abstract

PLA nanofibers are of great interest in tissue engineering due to their biocompatibility and morphology; moreover, their physical properties can be tailored for long-lasting applications. One of the common and efficient methods to improve polymer properties and slow down their degradation is sol-gel covalent crosslinking. However, this method usually results in the formation of gels or films, which undervalues the advantages of nanofibers. Here, we describe a dual process sol-gel/electrospinning to improve the mechanical properties and stabilize the degradation of PLA scaffolds. For this purpose, we synthesized star-shaped PLAs and functionalized them with triethoxysilylpropyl groups (StarPLA-PTES) to covalently react during nanofibers formation. To achieve this, we evaluated the use of (1) a polymer diluent and (2) different molecular weights of StarPLA on electrospinnability, StarPLA-PTES condensation time and crosslinking efficiency. Our results show that the diluent allowed the fiber formation and reduced the condensation time, while the addition of low-molecular-weight StarPLA-PTES improved the crosslinking degree, resulting in stable matrices even after 6 months of degradation. Additionally, these materials showed biocompatibility and allowed the proliferation of fibroblasts. Overall, these results open the door to the fabrication of scaffolds with enhanced stability and prospective long-term applications.

## 1. Introduction

Polylactide (or poly(lactic acid) (PLA)) is a promising material for biomedical applications due to their biocompatibility, degradability and mechanical properties [1,2,3]. This aliphatic polyester has been used in a wide range of medical devices (implants, surgical sutures, stents, etc.), scaffolds for tissue regeneration and drug delivery systems [4,5,6,7]. Conventional PLA processing for such applications includes film casting, injection molding, blow molding and foaming; these techniques, however, quite often present some important drawbacks. For instance, casted PLA films tend to lack porosity, a critical issue since nutrient and oxygen transport to cells cannot be assured. Similarly, the creation of pores through solidification is challenged with injection molding [8]. Lastly, even if foams exhibit desirable porosity and morphology for tissue engineering [9], their formulation increases the hydrophilicity of PLA, which in turn can speed up its degradation in a predominantly hydrophilic medium [10]. Alternatively, different research groups have shown a growing interest in the use of electrospinning to manufacture PLA scaffolds [11,12,13]: the nanofibrous scaffolds produced by this technique have morphological and architectural characteristics similar to those of the natural extracellular matrix [14,15], e.g., a very high surface/volume ratio and high porosity with appropriate pore size [13].

In addition to choosing an efficient and rather facile method to process PLA, modulation of the final properties can widen its versatility and applicability. In order to tune the mechanical properties and the degradation rate of PLA materials, many strategies have been used at the molecular level, including copolymerization, blending or crosslinking [16,17,18]. Among these, only crosslinking allows the formation of covalent bonds of PLA homopolymers, which would result in a strong and stable 3D network while keeping their inherent physicochemical properties. This approach can be classified into two groups: chemical crosslinking and crosslinking by exposure to low energy light or ionizing radiation [19,20]. Chemical crosslinking allows better control over the structure of the resulting materials due to the specificity of the reactive groups (crosslinkers), while irradiation, even if it is a solvent-free method (thus performed in softer conditions), normally leads to random crosslinking distributions [16,17]. There are two main paths to chemically crosslink PLA: reaction of peroxides on non-functional PLA of high-molecular-weight [21,22,23] and addition (or condensation) reactions of functionalized PLA oligomers [24,25], which results in stronger bonding (with no modification of PLA chains) compared to the use of peroxide.

While chemical crosslinking is regarded as a useful tool to enhance the physical features of PLA, cytotoxicity remains as a legitimate concern when the final material has prospective applications for health. In order to preserve the cytocompatibility of crosslinked PLA, two conditions have to be met: (1) crosslinkers grafted on PLA oligomers must react ideally without the use of catalysts and (2) no toxic by-products should be formed as result of this process. To this end, the crosslinking of siloxane functionalized PLA via sol-gel has been proposed as it offers chemoselectivity and mild reaction conditions [26,27,28]. We previously demonstrated that hydrolysis and condensation of alkoxysilane-bearing polymers could render, in soft conditions, modulable hydrogel or film networks linked through Si-O-Si bonds [29,30,31,32]. This method is based on two main steps: hydrolysis of the alkoxysilanes attached to the polymer to form silanol groups and condensation of the latter to form siloxane bonds. The combination of electrospinning and sol-gel process is rarely used but has been described, especially for the fabrication of ceramic or titanium oxide hybrid nanofibers [33,34,35,36]. These nanofibers are then made from TEOS “TetraEthyl Ortho Silicate” which reacts in a sol-gel process by trapping the elements without integrating them in the network. Other works also describe the use of water-soluble polymers such as PVA in such processes to facilitate electrospinning while allowing the sol-gel reaction of TEOS [37,38,39]. Very few studies use functionalized polymers to react via sol-gel. While such polymers are described, most studies in the literature focus on gels or films [40,41,42], which limit the advantages and potential of PLA as a biomaterial.

In this study, we took up the challenge for the first time to induce the hydrolysis of silanol-bearing PLA, followed by the condensation of reactive groups during (in situ) electrospinning to obtain crosslinked and stable nanofibrous scaffolds for medical applications.

In our research, we chose to work with 4-arm functionalized PLA in order to increase the number of reactive extremities, and therefore to promote the formation of siloxane bonds during electrospinning, facilitating the creation of a 3D network. We also sought to optimize the condensation of siloxane bonds by (i) introducing a diluent polymer that promotes the formation of electrospun fibers and (ii) varying the molecular weight (hence the arm size) of the star-shaped PLA in the electrospinning solution. The efficiency of the condensation was evaluated by quantifying the gel fraction and the degradation profile. Finally, the cytocompatibility and cell proliferation of the crosslinked nanofibers were also tested.

## 2. Results and Discussion

The main challenge in our study was to initiate the sol-gel reaction during the electrospinning process while allowing the formation of PLA nanofibers for the creation of a stable 3D polymeric network in a single step which, to the best of our knowledge, has never been reported in the literature. The creation of the network between the StarPLA-PTES is based on a hydrolysis of the triethoxysilane (SiOEt_3_) groups of PTES in an acidic environment which create silanol groups in the electrospinning syringe. Then, the silanol groups condensed with each other mainly during the travel time between the charged syringe and collector. For this purpose, StarPLAs were selected to increase the number of reactive chain ends, and promote their condensation, polymer entanglement and network formation as already demonstrated [15].

### 2.1. Synthesis and Functionalization of StarPLA

**Figure 1:**
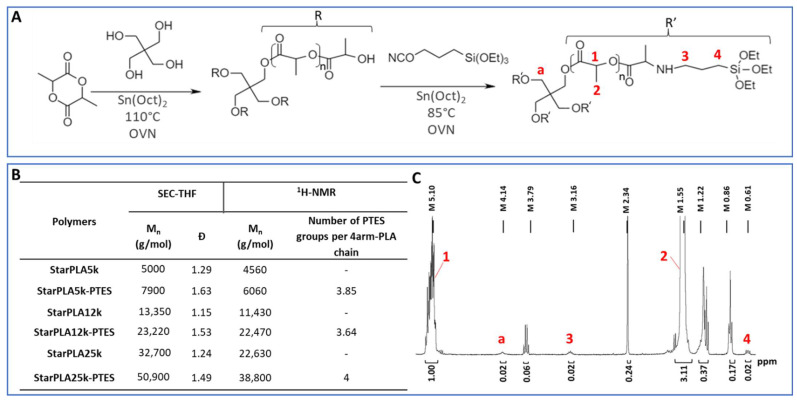
Synthesis and characterization of StarPLA and tetra(triethoxysilyl) StarPLA: (**A**) General synthesis and functionalization reactions; (**B**) ^1^H-NMR and SEC characterization of StarPLA and tetrafunctionalized StarPLA; (**C**) ^1^H-NMR spectrum of StarPLA25k-PTES.

The synthesis of the tetrafunctional StarPLAs is presented in Figure 1A. StarPLAs were obtained by polymerizing D,L-lactide onto 4-arm pentaerythritol by ring opening polymerization with Sn(Oct)_2_ as catalyst. We produced three different StarPLAs (StarPLA5k, StarPLA12k and StarPLA25k) with different lengths of PLA arms (Figure 1B). ^1^H-NMR analysis showed good agreement between theoretical and experimental molecular weights (Mn), suggesting that no transesterification reactions occurred. Mn values obtained by SEC were higher than those calculated by ^1^H-NMR, especially for StarPLA25k; this difference is however, explained by the SEC conditions (calibration, solvent) which render results that are rather relative for a particular polymer. All the synthesized star polymers presented (1) a narrow molecular weight distribution, with dispersity (Ð) values ranging from 1.15 to 1.63 and (2) a monodisperse profile, which is consistent with the synthesis of this type of polymer structure [43,44].

Then, the tetrafunctionalized StarPLA were obtained by reacting the isocyanate group of PTES with the hydroxyl groups at the chain end of the StarPLAs, resulting in the formation of a urethane bond in the presence of Sn(Oct)_2_. The degree of functionalization of PLA was calculated from ^1^H-NMR spectroscopy using the ratio between the methylene proton of the lactic units at 5.1 ppm and the methylene protons of triethoxysilane at 0.6 ppm (Figure 1C). The number of grafted PTES functions per 4-arm polymers was 3.85, 3.64 and 4 for StarPLA5k-PTES, StarPLA12k-PTES and StarPLA25k-PTES, respectively, which corresponds to a degree of functionalization of 94.5%, 91% and 100%, respectively.

### 2.2. Creation of StarPLA-PTES Nanofibers by Sol-Gel Process during Electrospinning

In a previous study, we showed the feasibility of producing two-dimensional polymer networks based on StarPLA-PTES [31]. Briefly, we triggered the hydrolysis of the siloxane functions of the polymer in solution and then poured it into a mold; the subsequent evaporation of the solvent caused the silanol groups to condense, leading to the formation of a crosslinked network. We aim here to fabricate nanofibrillar scaffolds made of StarPLA-PTES using electrospinning by triggering (i) the hydrolysis of PTES groups and (ii) the condensation of silanol groups (enhanced by solvent evaporation during polymer fiber ejection and stretching) within the same nanofiber-forming process.

**Figure 2:**
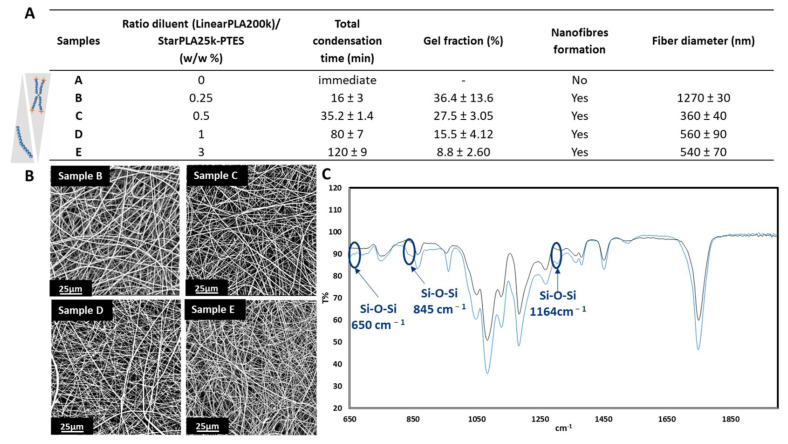
Crosslinking of nanofibers during electrospinning: (**A**) Polymer solution total condensation time, nanofibers gel fraction and fiber diameter; (**B**) SEM images of samples B, C, D and E; (**C**) FT-IR analysis of sample C without and with activation of the sol-gel reaction.

Among the different parameters used to evaluate the suitability of this double process, we considered the electrospinnability window as a crucial one. This parameter is the time required for the condensation of the silanol functions to alter the fluidity of the polymer solution such that it is no longer possible to electrospin it. We first started by studying the electrospinnability of StarPLA25k-PTES, due to its relevant molecular weight that would promote the polymer chain entanglements and formation of electrospun nanofibers. The hydrolysis of ethoxy groups was activated prior to electrospinning by introducing 10 µL of HCL (0.1 M in ethanol) per mL of polymers solution without any diluent (Figure 2A, Sample A). We observed that the condensation of the PTES functions into Si-O-Si bonds was very fast and generated immediately a gel in the syringe demonstrating that the macromolecular network was formed even before the solution could be electrospun. This very short gelation time is not generally found for nanofibers made from TEOS. TEOS, being a very short chain molecule, requires a long ageing time (between 1 to 4 h) [37,38] for the hydrolysis/condensation to generate a solution of sufficient viscosity to be electrospun. In order to slow down the condensation time and allow the electrospinning process to occur, we used a linear PLA of 200,000 g·mol^−1^ as a diluent polymer to serve as a spacer between the reactive species and we evaluated its impact on the formation of fibers, the condensation time and the gel fraction. For this purpose, we varied the diluent/StarPLA25k-PTES mass ratio by 0.25, 0.5, 1 and 3 (Figure 2A, samples B, C, D and E respectively). In order to compare the samples with each other, we kept the electrospinning parameters unvaried during their fabrication. In contrast to the mixture without diluent polymer (sample A), the four samples containing the diluent increased the window of electrospinnability and thus permitted the generation of nanofibers that were free of defects (Figure 2B). As expected, the hydrolyzed functions were distanced from each other and this changed according to the diluent/star polymer ratio used. For instance, the total condensation time of mixture B, which contained 1/4 diluent, was 16 min and it increased to 120 min when the mass of diluent was three times bigger than that of the star polymer (mixture E). In terms of nanostructure, all the electrospun fibers were in the range of 360 to 560 nm, except for the 0.25 diluent/star polymer ratio (1.27 µm). In all the cases, the fiber size distribution was found to be homogeneous, which was confirmed by their small standard deviation (Figure 2A).

In order to confirm the condensation of PTES groups during electrospinning, we analyzed the obtained nanofibers by FT-IR. The results show the condensation of Si-OH groups into Si-O-Si in crosslinked samples (when hydrolysis was initiated) compared to non-crosslinked ones (no hydrolysis), by the appearance of signals at 1164 cm^−1^, 845 cm^−1^ and 650 cm^−1^ (Figure 2C). It is well known that a crosslinked material is insoluble in a solvent that solubilizes it when not crosslinked. Thus, we qualitatively tested the nanofibers solubility in THF (good solvent for StarPLAs) and we noticed that the fibers of samples B and C were insoluble; sample D was partially soluble while sample E was totally dissolved in such solvent. Next, we evaluated the degree of crosslinking: we measured the gel fraction, which corresponds to the percentage of material actually crosslinked and therefore not solubilized by an organic solvent. This confirmed that the increase in the diluent/StarPLA25k-PTES ratio results in a decrease of network formation: we obtained gel fractions of 36.4, 27.5, 15.5 and 8.8% for diluent/StarPLA25k-PTES ratios of 0.25, 0.5, 1 and 3 (samples B to E, respectively). In summary, as the amount of polymer diluent in the solution increases (thus provoking more steric hindrance), the time required for the condensation of the PTES functions increases (i.e., longer electrospinnability window) but the efficiency of the crosslinking is, importantly, reduced.

**Figure 3:**
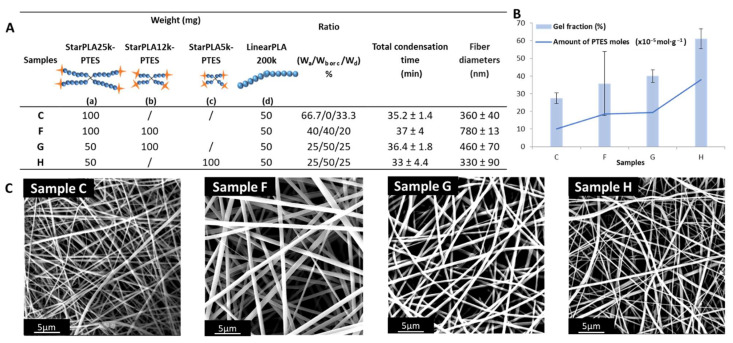
Effect of the density of PTES groups on the crosslinking efficiency of the nanofiber network: (**A**) Influence of molecular weight of the added polymer on the total condensation time and fiber diameter; (**B**) Evolution of the gel fraction as a function of the density of PTES groups; (**C**) SEM images of samples C, F, G and H.

In order to maximize the network formation during electrospinning, we increased the density of PTES functions in sample C by introducing low-molecular-weight tetrafunctionalized polymers (StarPLA12k-PTES and StarPLA5k-PTES). We chose sample C because it represents a good compromise between the time needed to obtain a nanofiber layer (driven by the condensation time) and the degree of crosslinking of the nanofibers (given by the gel fraction). First, we added StarPLA12k-PTES to an equivalent amount of StarPLA25k-PTES to form sample F (Figure 3A). This increase of the number of PTES moles from 10.1 to 18.4 × 10^−5^ mol·g^−1^ slightly enhanced the gel fraction from 27.5 to 35.7%, although a large variability was also obtained (Figure 3B).

To evaluate the influence of the length of StarPLA arms without significantly modifying the amount of PTES moles and in view of reducing the standard deviation between the gel fractions obtained, we promoted the predominance of StarPLA12k-PTES in the mixture by halving the mass quantity of StarPLA25k-PTES contained in the sample F (which resulted in sample G). The gel fraction of the nanofibers obtained from sample G was increased by 4% compared to the mixture F with no change in the condensation time.

Finally, to further improve the crosslinking rate, we replaced StarPLA12k-PTES with the low-molecular-weight StarPLA5k-PTES (sample H), keeping the same weight ratio as in sample G. This rendered a significant increase in the gel fraction from 40.0 to 61.1%. This enhancement in the condensation of silanol groups by the addition of low-molecular-weight polymers was directly correlated to the increase in the density of the crosslinking groups in solution (i.e., the polymer/functional groups ratio decreases with shorter chains). Moreover, the improvement of the gel fraction and thus the creation of a polymer network in samples G and H (which is also confirmed with the insolubility of the fibers in THF) did not affect the electrospinnability window (~35 min), which is the time needed to obtain scaffolds that are sufficiently stable for handling in our case. We believe this conservation of the electrospinnability window is related to the low viscosity of the solutions due to the use of short chain length polymers [45], which creates space between the reactive functions in comparison with polymers of longer chains, which induce a longer time to generate the condensation.

The resulting nanofibers had diameters between 360 and 780 nm (Figure 3A,C). This is a significant reduction of the fiber size by adding low-molecular-weight polymers. Indeed, the shorter the chain length of the polymer arm, the finer the fiber diameter. This phenomenon is explained by the decrease in the solution viscosity due to low-molecular-weight polymers (given by the Mark–Houwink equation), which in turn has a direct impact on the fiber size [46,47].

### 2.3. Nanofibers Behavior in a Physiological Environment-Like over Time

Hydrolysis is the primary mechanism for degradation of aliphatic polyesters [48,49]. In this study, we investigated the influence of the sol-gel process and the formation of siloxane bonds on the rate of hydrolytic degradation of nanofibers. In this context, we tracked the evolution of degradation over 6 months of samples G and H (the most promising ones in terms of resulting gel fraction), and compared them to the reference sample C, which is composed of only one type of functionalized StarPLA, in order to assess the influence of the crosslinking group density on the hydrolysis of the scaffolds (Table 1).

For both C and G scaffolds, the gel fraction increased in the first month of degradation from 27.5 to 76.2% and from 40 to 85.2%, respectively, and stabilized for the remaining months. For scaffold H, the gel fraction did not change during the whole degradation period. The increase in the gel fraction for C and G can be attributed to an increase in hydrolysis reactions of unreacted PTES groups during electrospinning, which subsequently condensed. The following stabilization is then related to a total consumption of the functions (i.e., a saturation of crosslinked groups). In the case of scaffold H, this saturation is highly likely to have been reached during the same electrospinning process, hence the unchanged values of gel fraction from the beginning of the degradation period. The reconstituted bonds during degradation, together with the bonds already present, led to the formation of a stable covalent network in the nanofibers, which explains the low mass loss during the 6 months. Arsenie et al. also reported that the degradation of a StarPLA-PTES film crosslinked with siloxane bonds was stable during their degradation assay period (8 weeks) [15].

In contrast, non-crosslinked StarPDLAs degrade rapidly: it has been shown that 6-arm StarPLAs films can lose up to 18% of their mass in 45 days [50] which might be disadvantageous for prolonged use. The stiffness of the scaffolds was also preserved during the whole degradation period. Indeed, after 6 months the Young’s modulus only (and slightly) evolved for H nanofibers (from 20.8 ± 4 to 37.1 ± 11 Mpa), while for C and G nanofibers it remained practically unchanged (from 19.1 ± 5 to 19.8 ± 8 Mpa and from 37.9 ± 19 to 35.2 ± 3 Mpa, respectively).

**Figure 4:**
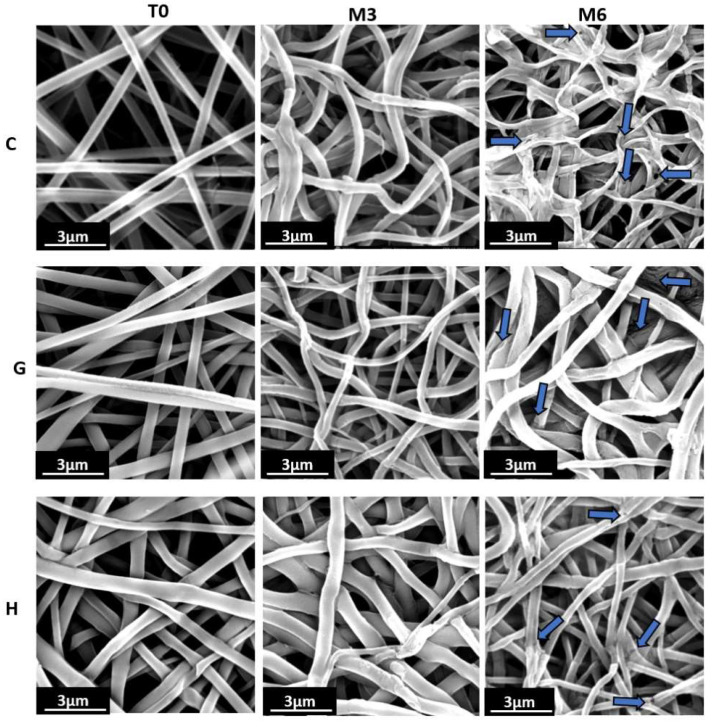
Morphology of samples C, G and H before (T0) and after 3 and 6 months of degradation. The blue arrows show degradation cracks on the surface of nanofibers at month six.

Although the properties of the nanofibrillar scaffolds were stable overall, SEM images showed (i) the appearance of some cracks or fissures on the G and H nanofibers and (ii) the beginning of loss of shape of the C nanofibers, suggesting that degradation was starting after 6 months (Figure 4).

### 2.4. Biological Evaluation In Vitro

**Figure 5:**
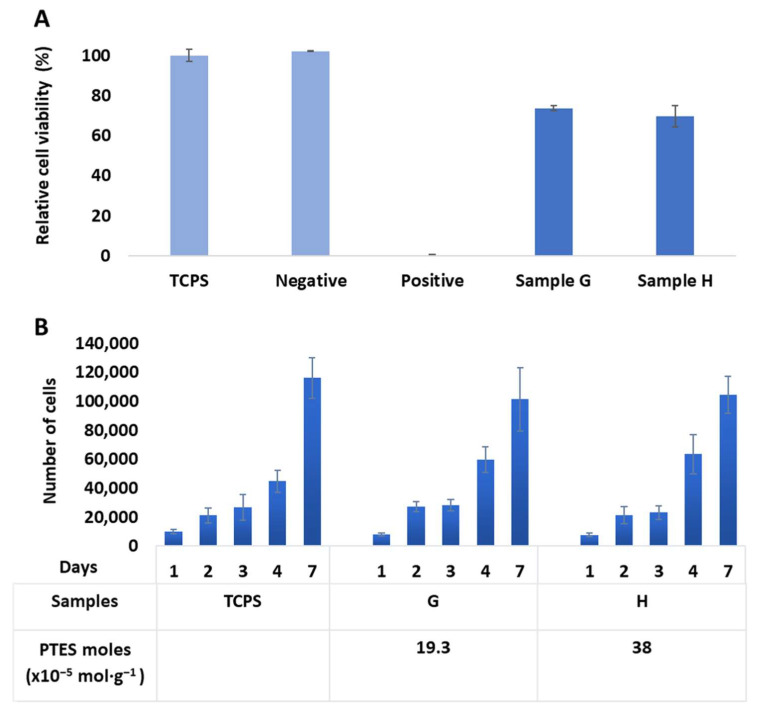
Biological evaluation in vitro: (**A**) Samples G and H were not cytotoxic after 24 h of contact with L929 cells compared to TCPS (tissue culture polystyrene); negative and positive controls were also tested (high density polyethylene film and polyurethane film + 0.1% zinc diethyldithiocarbamate, respectively). (**B**) The proliferation of L929 cells on G and H nanofibers was similar to that on TCPS over 7 days (*p* > 0.05).

While the biocompatibility of PLA is widely mentioned in the literature [3,51], we wanted to evaluate whether the fabrication of StarPLA-PTES nanofibers as well as the presence of covalent crosslinking in the final network impart any cytotoxic effects in the final material, for potential use in tissue engineering (hence the use of L929 cell line as target). To this end, we selected mixtures G and H, corresponding to a PTES density of 19.3 and 38 × 10^−5^ mol·g^−1^. Compared to the viability on TCPS control, the results show a L929 viability of 70% and 74% for nanofibers G and H, respectively (Figure 5A). These are acceptable cell survival levels according to the ISO 10993-5 norm.

We next evaluated the L929 cell proliferation on the same samples over 7 days (Figure 5B). The results show that there is no significant difference between the proliferation on the two nanofibers and on TCPS control, with a calculated *p* value between 0.07 and 0.9 for any given time point. Thus, we can conclude that the presence of Si-O-Si bonds (at the levels used in this study) did not negatively influence the proliferation of L929. Overall, these studies show that the nanofibers obtained were non-cytotoxic, permitted good cell proliferation and therefore are good candidates for future biomedical applications.

## 3. Materials and Methods

### 3.1. Materials

D,L-lactide was provided by Corbion (Gorinchem, The Netherlands). Tin (II) 2-ethylhexanoate (Sn(Oct)_2_, 95%), pentaerythritol, diethylene glycol (DEG), (3-isocyanatopropyl) triethoxysilane (IPTES), dichloromethane, heptane, toluene, tetrahydrofuran, hydrochloric acid (37%) and hydrochloric acid (37%) (1 M in methanol), dichloromethane (DCM), trifluoroethanol, trifluoroacetic acid and phosphate buffer solution (PBS) were purchased from Sigma-Aldrich (St Quentin Fallavier, France).

CellTiter Glo assay was provided by Promega G7571 (Charbonnières-les-Bains, France). PrestoBlue^®^ assay (A13262) and Clariostar plate reader (A13626) were acquired from Invitrogen (Illkirch, France). Negative RM-C and positive RM-A were supplied by Hatano Research Intitue, Food and Drug Safety Center, Hadano, Japan).

### 3.2. Synthesis of Linear and 4-Arm Star Poly(Lactide)

We synthesized LinearPLA of 200 kg·mol^−1^ and 4-arms StarPLA of 25 kg·mol^−1^, 12 kg·mol^−1^ and 5 kg·mol^−1^, i.e., LinearPLA200k, StarPLA25k, StarPLA12k and StarPLA5k, respectively. Polymers were synthesized by ring opening polymerization, using a procedure previously described [31]. Typically, for the synthesis of StarPLA-25k: D,L-lactide (30 g, 208 mmol, 14 eq), pentaerythritol as multifunctional initiator (0.163 g, 1.19 mmol, 1 eq) and SnOct_2_ as catalyst (0.194 g, 4.79 mmol, 0.4 eq) were introduced in a flask. After 2 h under vacuum, the flask was sealed and maintained at 120 °C for five days. The obtained polymers were solubilized in DCM and then precipitated in cold heptane (4 °C). Finally, the recovered polymer was vacuum dried overnight. The average reaction yields were 88 ± 9%. StarPLA12k and StarPLA5k were obtained by varying the molar ratio monomers/initiator: 20.8 and 8.67 respectively. The linearPLA200k was synthesized with the same protocol of polymerization of the StarPLA, but this time using diethylene glycol as initiator in a monomer/initiator molar ratio of 694.4. The molecular weight of the StarPLAs was determined by ^1^H-NMR from the signal ratio between methylene protons of pentaerythritol and the methyl proton of the lactic unit.

^1^H-NMR (400 MHz, CDCl_3_) δ (ppm) = 5.15 (m, C**H**CH_3_); 4.34 (m, C**H**CH_3_OH); 4.14 (s, CC**H**_3_O); 3.5 (s, CC**H**_3_OH); 1.54 (m, CHC**H**_3_)

### 3.3. Functionalization of 4-Arm Starpoly(Lactide) with Triethoxysilane

Polymers were functionalized using a procedure previously described [31]. Typically, the StarPLA25k (5 g, 2.5 mmol) was put in a flask under vacuum for 4 h. The polymer was solubilized in anhydrous toluene (100 mL) under inert gas during 1 h. Then, the IPTES (3.71 g, 15 mmol, 6 eq) was added in the solution and SnOct_2_ as catalyst (0.162 g, 0.4 mmol, 0.16 eq). The reaction was carried out at 75 °C, with constant stirring and under inert atmosphere for 24 h. The obtained StarPLA25k-PTES was purified in cold heptane and vacuum dried in order to eliminate traces of solvent. StarPLA12k and StarPLA5k were functionalized using the same protocol as for StarPLA25k.

The degree of functionalization was determined by ^1^H-NMR from the signal ratio between the methyl lactic proton (δ = 5.1 ppm) and the methylene proton linked to triethoxysilane (δ = 0.6 ppm).

^1^H-NMR (400 MHz, CDCl_3_) δ(ppm) = 5.15(m, CHCH_3_); 4.34(m, CHCH_3_OH); 4.14 (s, CCH_3_O); 3.83 (m, OCH_2_CH_3_); 3.5 (s, CCH_3_OH); 3.17 (m, NHCH_2_); 1.54 (m, CHC**H**_3_); 1.2 (m, OCH_2_C**H**_3_); 0.64–0.54 (m, CH_2_C**H**_2_Si).

### 3.4. Electrospinning

StarPLA-PTES were dissolved in TFE at concentrations ranging from 15 to 25 wt%. In order to activate the sol gel process, 10 µL of HCL (0.1 M in ethanol) was added per mL of polymer solution. After one minute of agitation using a vortex, the mixture was loaded into a syringe with a 21-G needle and fixed on a syringe pump (KDS100, KD Scientific,). To start the electrospinning process, a voltage of 15 kV was applied to the polymer solution, which was dispensed at a flow rate of 0.5 mL·h^−1^. The nanofibers were collected on a flat collector that was placed 15 cm from the syringe needle. The experiments were performed at 25 ± 1 °C with a relative humidity of 48 ± 3%. All the tests were carried out under the same conditions.

### 3.5. Characterization Methods

Size exclusion chromatography (SEC) was conducted using a Shimadzu LC-200AD Prominence system (Shimadzu, Marne-la-Vallée, France) equipped with a PLgel MIXED-C guard column (Agilent, 5 μm, 50 mm length × 7.5 mm diameter), two PLgel MIXED-C columns (Agilent, 5 μm, 300 mm length × 7.5 mm diameter) and a RID-20A refractive index signal, using poly (ethylene glycol) as calibration standard with a flow rate = 1 mL·min^−1^. The polymer was solubilized in THF at 10 mg·mL^−1^; 200 µL of this solution was injected in the system. ^1^H-NMR measurements were carried out at 300 MHz with an AMX300 Bruker spectrophotometer (Cambridge Scientific Products, Watertown, USA) at room temperature using deuterated chloroform as solvent. Fourier transform infrared analysis was performed using a Perkin Elmer Spectrum 100 (PerkinElmer France, Villebon-sur-Yvette, France); four scans were performed at room temperature in the range of 4000–650 cm^−1^.

For scanning electron microscopy (SEM), the samples were sputter coated with a 10 nm (2 min) thick gold film and imaged under a scanning electron microscope (Phenom-world ProX) using an accelerating voltage of 15 kV. Micrographs were analyzed with ImageJ (https://imagej.nih.gov/ij/download.html (accessed on 12 April 2021)). The diameter of the fibers was measured with the same software by drawing a perpendicular line on both edges of the fiber (number of fibers measured for each condition = 50).

The differential scanning calorimetry (DSC) analysis was conducted under argon with a Mettler Toledo 3 Star DSC system. A total of 3 mg of each nanofiber sample was weighed into a standard aluminum dish. The thermal cycle consisted of a heating sweep from 0 °C to 200 °C (10°C·min^−1^) followed by cooling to −10 °C (10°C·min^−1^) and a second heating sweep to 300 °C (10°C·min^−1^). The glass transition temperature (Tg) was measured on the first ramp of heating.

### 3.6. Gel Fraction

For each condition, gel fraction was measured by weighing 3 samples (w_i_) followed by immersion in 3 mL of DCM under agitation for 3 h. Next, the DCM solution was centrifugated (3000 rpm for 10 min) to separate the insoluble and soluble fractions. After drying under vacuum for 24h, the insoluble fractions were weighed (w_insoluble_). Gel fraction was calculated with Equation (1) after verifying that the addition of soluble and insoluble phases was 100% of the initial sample weight:

Gel fraction (%) = (w_insoluble_ /w_i_) × 100 (1)

### 3.7. Tensile Tests

Mechanical properties were measured on C, G, H samples (3 cm wide × 1 cm length, n = 4) after different degradation time points, using uniaxial tensile testing on an Instron 3344 testing system at a speed of 5 mm·min^−1^ and at 37 ± 1 °C. The Young’s modulus (E, MPa) was calculated based on the initial linear section of the stress–strain curve and was reported as the mean value of the measurements.

### 3.8. Nanofibers Behavior in a Physiological Environment-Like over Time

Electrospun scaffolds (10 mm length × 30 mm wide, 7.5 ± 3 mg) were weighed (m_i_ = initial mass) and then immersed in 6 mL of PBS (pH = 7.4) at 37 °C under constant stirring. Samples were removed from PBS at months one, three and six and their mass loss, gel fraction and mechanical properties were evaluated using quadruplicates.

The water uptake and mass loss were calculated from Equations (2) and (3), respectively:

Water uptake (%) = ((m_w_ − m_i_)/m_i_) × 100 (2)

Mass loss (%) = ((m_i_ − m_d_)/m_i_) × 100 (3)

where m_w_ is the hydrated mass of the scaffolds and m_d_ is mass after vacuum drying overnight.

The gel fraction and mechanical properties of degraded samples were evaluated following protocols described in Section 3.6 and Section 3.7, respectively.

### 3.9. Biological Evaluation In Vitro

NCTC-Clone 929 cells (mouse fibroblast cell line (ECACC 85011425)), passage 32, were cultured in 500 mL MEM with 5 mL glutamax (1% stabilized glutamine), 50 mL horse serum, and 100 U/mL penicillin and streptomycin 100 μg/mL.

#### 3.9.1. Cytotoxicity Assay

The in vitro cytocompatibility of scaffolds was tested following EN ISO 10993-5 standard protocols (n = 3). Samples were irradiated with UV-C (λ = 254 nm, 5 min, 80 W) for decontamination. Then, 1.5 mL of complete cell culture medium (DMEM 4.5 g/L D-glucose supplemented with 5% FBS, 1 mM L-glutamine, 100 U/mL penicillin and 100 µg/mL streptomycin) was added to the scaffolds and kept at 37 °C under constant stirring for 72 h. Next, 0.1 mL of this extracting medium was added to murine fibroblasts L929 P32 previously seeded at 1.10^4^ cells per well in a 96-well plate the night before to allow their adhesion. After 24 h of incubation at 37 °C in a humid environment, cell viability was assessed based on ATP quantification using CellTiter Glo (Promega G7571) according to the supplier instructions. Briefly, 50 µL of media was removed from each well, then 50 µL of CellTiter Glo reagent was immediately added to each well containing the cells and incubated for 10 min in the dark. Finally, luminescence was read and the results for each sample were compared to TCPS. Negative (high-density polyethylene film) and positive (polyurethane containing 0.1% zinc diethyldithiocarbamate) controls were also tested.

#### 3.9.2. Proliferation Assay

Samples (n = 4) were cut into disks of 2 cm diameter, sterilized with UV-C irradiation (λ = 254 nm, 2.5 min on each side, 80 W) and placed in non-treated 24-well plates. Scaffolds were kept in a fixed position with the use of O-rings. L929 cells were seeded on top of the scaffolds at 8.10^4^ cells per well and were placed at 37 °C and 5% CO_2._. The number of cells was assessed after 1, 2, 3, 4 and 7 days of contact with the scaffolds using a PrestoBue assay that evaluates the transformation of weakly fluorescent blue resazurin into highly fluorescent red resorufin through the mitochondrial activity of the cell. Briefly, this reagent was placed at a volume ratio of 1:10 in each well and incubated in the dark for 40 min at 37 °C. Fluorescence intensity was then read in a CLARIOstar^®^ microplate reader (wavelength: excitation 558 nm, emission 590 nm). After each measurement, the supernatant was replaced with fresh medium to continue the cell culture until day 7.

#### 3.9.3. Statistical Analysis

R software version 3.5.2 (R Foundation, Vienna, Austria) was used to perform statistical analysis. Significance was assessed by non-parametric Kruskall–Wallis test with repeated measures followed by Dunn’s post-test. Values of *p* > 0.05 were considered as not statistically significant.

## 4. Conclusions

Electrospun PLA scaffolds are of great interest in biomedical applications, not only because of their biocompatibility and nanostructure (which resembles the extracellular matrix) but also because they display appropriate degradation times for tissue engineering. In this study, we created a crosslinked PLA network to further extend the degradation time of such scaffolds. We demonstrated that it is possible to create a stable and slow degrading 3D network by activating the sol-gel process during electrospinning. The presence of a polymer diluent was needed to allow the formation of fibers; the crosslinking degree of the network (as well as the final properties) could be modulated by adjusting the proportion of diluent and the molecular weight of the star polymers in the mixture. In addition, these nanofibrillar materials were not cytotoxic and allowed the proliferation of L929 cell line. Future studies will include the incorporation of bioactive peptides (functionalized with IPTES) into the StarPLA network, using our condensation method to fabricate nanofibrillar structures that are not only stable over long periods, but that also trigger a biological response in cells.

## Figures and Tables

**Table 1:** Scaffold properties during 6 months of degradation in vitro.

Samples	Degradation Time (Months)	Gel Fraction (%)	Mass Loss (%)	Young Modulus E (Mpa)	Glass Transition (°C)
**C**	0	27.5 ± 3	/	19.1 ± 5	60.7
	1	76.2 ± 2	5.5 ± 4	27.8 ± 7	49.5
	3	80 ± 3	0	23 ± 1	52.6
	6	84.7 ± 5	7.9 ± 5	19.8 ± 8	54.2
**G**	0	40 ± 4	/	37.9 ± 19	54.8
	1	85.2 ± 9	5.8 ± 4	31.5 ± 9	48.3
	3	81.8 ± 1	1.2 ± 2.1	82.3 ± 7	48.4
	6	81.9 ± 7	1.7 ± 0.6	35.2 ± 3	48
**H**	0	61 ± 6	/	20.8 ± 4	52.1
	1	65.3 ± 14	8.7 ± 7	20.3 ± 3	48.8
	3	65.7 ± 3	1.5 ± 2.2	32.8 ± 11	50
	6	72.3 ± 5	1.0 ± 1	37.1 ± 11	48

## Data Availability

The data presented in this study are available on request from the corresponding author.

## Sample Availability

All samples are available from the authors.
